# Semantics-based composition of EMBOSS services

**DOI:** 10.1186/2041-1480-2-S1-S5

**Published:** 2011-03-07

**Authors:** Anna-Lena Lamprecht, Stefan Naujokat, Tiziana Margaria, Bernhard Steffen

**Affiliations:** 1 Chair for Programming Systems, Technical University Dortmund, Dortmund, D-44227, Germany; 2 Chair for Service and Software Engineering, Potsdam University, Potsdam, D-14482, Germany

## Abstract

**Background:**

More than in other domains the heterogeneous services world in bioinformatics demands for a methodology to classify and relate resources in a both human and machine accessible manner. The Semantic Web, which is meant to address exactly this challenge, is currently one of the most ambitious projects in computer science. Collective efforts within the community have already led to a basis of standards for semantic service descriptions and meta-information. In combination with process synthesis and planning methods, such knowledge about types and services can facilitate the automatic composition of workflows for particular research questions.

**Results:**

In this study we apply the synthesis methodology that is available in the Bio-jETI workflow management framework for the semantics-based composition of EMBOSS services. EMBOSS (European Molecular Biology Open Software Suite) is a collection of 350 tools (March 2010) for various sequence analysis tasks, and thus a rich source of services and types that imply comprehensive domain models for planning and synthesis approaches. We use and compare two different setups of our EMBOSS synthesis domain: 1) a manually defined domain setup where an intuitive, high-level, semantically meaningful nomenclature is applied to describe the input/output behavior of the single EMBOSS tools and their classifications, and 2) a domain setup where this information has been automatically derived from the EMBOSS Ajax Command Definition (ACD) files and the EMBRACE Data and Methods ontology (EDAM). Our experiments demonstrate that these domain models in combination with our synthesis methodology greatly simplify working with the large, heterogeneous, and hence manually intractable EMBOSS collection. However, they also show that with the information that can be derived from the (current) ACD files and EDAM ontology alone, some essential connections between services can not be recognized.

**Conclusions:**

Our results show that adequate domain modeling requires to incorporate as much domain knowledge as possible, far beyond the mere technical aspects of the different types and services. Finding or defining semantically appropriate service and type descriptions is a difficult task, but the bioinformatics community appears to be on the right track towards a Life Science Semantic Web, which will eventually allow automatic service composition methods to unfold their full potential.

## Background

Research projects in modern molecular biology rely on increasingly complex combinations of computational methods to handle the data that is produced in the life science laboratories. The plethora and kind of data involved in modern research in the field of biology is only accessible by computational methods. Bioinformatics algorithms, tools, and databases, are available in various ways, developed by different groups, in different contexts, using different technologies. The abundance of heterogeneous resources provided by different institutes all over the world leads to the problem of finding the right service for a certain task. The Semantic Web [1] aims at thoroughly equipping individual data and services with machine-processable meta-information in order to simplify the discovery of relevant resources. The importance of properly semantically annotated data and services has been recognized by the life science community earlier than by other application domains, and thus various projects have made significant progress towards a Semantic Web for bioinformatics [2]. Making no claim to be complete, the following list of projects characterizes the current state of the art:

● BioMoby [3] is an open bioinformatics web services registry, which particularly started the modeling of the bioinformatics domain. Making use of service and type meta-data and ontologies for classifying them further, a number of services has been prepared mainly for supporting semantics-based retrieval. However, the native Moby specifications originate from the early 2000s and thus do not adhere to the Semantic Web standards, which have been developed in the last years, but on self-made realizations of the same concepts.

● The SADI (Semantic Automated Discovery and Integration) [4] framework provides an open service registry that, in contrast to its predecessor BioMoby, uses standards-compliant Semantic Web Service design patterns to deploy and operate bioinformatics web services. In addition to the collection of services, a simple OWL-based ontology is available that classifies the heterogeneous resources further.

● The BioCatalogue [5] is a recently released, curated registry for life science web services. It provides a comprehensive portal for discovering, registering, annotating and monitoring services that also makes extensive use of different Web 2.0 community features, like collaborative tagging of services and various newsfeeds.

● The myGrid ontology [6] is one of the sources of information that the BioCatalogue uses. It has been developed with the aim of supporting service discovery. It consists of two parts, namely the service ontology and the domain ontology. The former describes the physical and operational features of web services (e.g., inputs and outputs), while the latter captures descriptions of bioinformatics data types and their relationships.

● The EMBRACE Ontology for Data and Methods (EDAM, [7]) is an ontology for bioinformatics tools and data, which aims at providing a controlled vocabulary for the diverse services and resources in the Life Science Semantic Web.

The challenge of semantics-based service composition in the bioinformatics application domain has been addressed by a number of projects. For instance, the BioMoby project provides a composition functionality for its services: with the MOBY-S Web Service Browser [8] it is possible to search for an appropriate next service and store the sequence of executed tools as a Taverna [9] workflow. Similarly, the REMORA web server [10] offers functionality for the discovery and step-by-step composition of BioMoby services and the DDBJ’s Web API for biology provides next applicable services according to the outputs of previously executed services [11]. Another example is the scenario presented in [12], where meaningful terms from the gene expression domain are recognized in the text of a web page and used for the formulation of higher-level goals, which are, together with web services that are linked to the terms, given to an HTN (Hierarchical Task Network) planner in order to create workflows that are suitable within the current context. All these have clear limitations, as their automatic service composition functionality is:

● restricted to small sub-workflows or even single steps of the workflow, which comes with the risk that users get stuck when stepwisely trying to construct the globally intended solution, 

● limited to semantically annotated services of the particular platform.

Current tools for the graphical development of bioinformatics workflows [9,13-16], most of them data-flow based, do not include means for semantic modeling or automatic service composition. An exception is Bio-jETI [17,18], which bridges this gap by supporting the incorporation of semantically modeled domain information for control-flow oriented process construction. Its holistic perspective covers both the process modeling and the integration of individual services and platforms:

● Process development is addressed from a goal-oriented global perspective. A loose programming concept allows the user to specify the actually intended workflow as a whole, and the synthesis finds shortest solutions directly matching the global intent.

● Service descriptions in terms of the domain model are decoupled from the technical service specifications and implementations, so that any kind of heterogeneous resource at any location can be integrated, and there is no restriction to semantically annotated services of a particular platform.

In this paper we extend a previous case study on the semantics-based composition of EMBOSS services with Bio-jETI [19]. We use two different setups, one manually defined and one automatically generated from available meta-information, and compare their characteristics and the respective synthesis results.

## Results and discussion

EMBOSS (European Molecular Biology Open Software Suite [20,21]) is a collection of freely available tools for the molecular biology user community. It contains a number of small and large programs for a wide range of tasks, such as sequence alignment, database searches, protein motif identification, nucleotide sequence pattern analysis, and codon usage analysis as well as the preparation of data for presentation and publication. As of March 2010, EMBOSS (Release 6.2.0) consists of around 350 tools, some derived from originally standalone packages.

EMBOSS provides a common technical interface for the diverse tools that are contained in the suite. They can be run from the command line, or accessed from other programs. Thus, EMBOSS is also suitable for being set up behind GUIs and web interfaces. What is more, it automatically copes with data in a variety of formats, even allowing for transparent retrieval of sequence data from the web. The EMBOSS tools work seamlessly for a number of different formats and types, and therefore free the user from caring about compatibility and type conflicts. This enables us to focus on the actual service semantics rather than on technical details of data compatibility when setting up the domain.

We give a detailed description of our synthesis method and its integration into the Bio-jETI framework (called PROPHETS) in the Methods section. PROPHETS supports domain-specific workflow modeling and synthesis in two principal phases:

1. Domain modeling.

2. Workflow design.

Roughly speaking, the domain modeling involves everything that is required prior to domain-specific workflow development, such as service integration and providing meta-information about the services and types of the application domain. The actual process modeling is then done by the workflow designer, who benefits from the domain model that has been set up according to his needs, referring to services and data types using familiar terminology. The workflow designer does not need to care about technical details like type consistency. He can mark the connection between certain services as* loosely specified,* thus leaving the problem of proper type conversion to the synthesis algorithm.

Starting from the beginning, setting up a domain for PROPHETS involves three major steps:

1. Integration of services.

2. Description of the input/output behavior of the individual services.

3. Structuring of the domain by classification of types and services in taxonomies (i.e. simple ontologies that relate entities in terms of* is-a* relations).

The integration of the EMBOSS services that we used in this study was done automatically. We let a script process the tool directories of the EMBOSS source code repository and create workflow building blocks for all available tools. In the following, we describe two disparate procedures that we used to set up synthesis domains for the EMBOSS suite, regarding the service descriptions and taxonomic classifications of types and services:

● * manually*, where intuitive, high-level, semantically meaningful nomenclature for types and services is provided by a domain modeler, and

●  *automatically,* where the information about types and services is derived from the EDAM Ontology and the EMBOSS Ajax Command Definition (ACD) files.

In the remainder of this section, we show by means of some workflow examples what the synthesis methodology can infer from these domains and where the principal differences are.

The graphical presentations of the domain (including all EMBOSS tools and several abstract groups) are not suited to be represented in paper page format. Therefore, we use a small subset of this domain for presentation in this paper. Table 1 lists the services in this subset along with a short description of their function. The subset consists mainly of the HMMER [22,23] applications. HMMER is a software for biosequence analysis using Profile Hidden Markov Models. It contributes 9 applications to EMBOSS, namely ehmmalign, ehmmbuild, ehmmcalibrate, ehmmconvert, ehmmemit, ehmmfetch, ehmmindex, ehmmpfam, and ehmmsearch. The prefix ’e’ is used to distinguish the EMBOSS integration from the orginal HMMER programs. In addition to the HMMER tools, the subset contains the multiple sequence analysis tools emma and edialign, makeprotseq and makenucseq for the generation of random protein and nucleotide sequences, respectively, as well as some tools for the display of specific data (showalign, showfeat, showpep, showseq).

**Table 1:** Services in the HMMER subset of the EMBOSS domain.

Service	Function
edialign	Local multiple alignment of sequences.
ehmmalign	Align sequences to an HMM profile.
ehmmbuild	Build a profile HMM from an alignment.
ehmmcalibrate	Calibrate HMM search statistics.
ehmmconvert	Convert between profile HMM file formats.
ehmmemit	Generate sequences from a profile HMM.
ehmmfetch	Retrieve an HMM from an HMM database.
ehmmindex	Create a binary SSI index for an HMM database.
ehmmpfam	Search one or more sequences against an HMM database.
ehmmsearch	Search sequence database with a profile HMM.
emma	Global multiple alignment of sequences.
makenucseq	Create random nucleotide sequences.
makeprotseq	Create random protein sequences.
showalign	Display a multiple sequence alignment in pretty format.
showfeat	Display features of a sequence in pretty format.
showpep	Displays protein sequences with features in pretty format.
showseq	Display sequences with features in pretty format.

This table lists the services in the HMMER subset of the EMBOSS domain and gives a short description of their function.

### Manual domain setup

As stated above, after the (mere technical) integration of the EMBOSS services into the framework, setting up the domain consists of describing the input/output behavior of the services and structuring services and data types by taxonomic classifications. In short, the manual setup procedure involved basically two steps:

1. Extracting information about input and output types from natural language documentations of the services.

2. Adding classifications of service and types based on further natural language documentations and own knowledge and experiences.

This manual setup for the EMBOSS synthesis domain originated from a former case study [19]. We applied natural language documentations of the services from different sources that are available on the web, primarily from the project web site’s list of EMBOSS [24] and EMBASSY [25] applications, and from the EBI’s EMBOSS web service descriptions in SoapLab [26]. Table 2 lists the services in the HMMER subset of the domain along with their input and output data types.

**Table 2:** Manually defined domain: services in the HMMER subset.

Service	Input Types	Output Types
edialign	MultipleSequence	Alignment
ehmmalign	HMM, Sequence	Alignment
ehmmbuild	Alignment	HMM
ehmmcalibrate	HMM	HMM
ehmmconvert	HMM	HMM
ehmmemit	HMM	EhmmemitOutput
ehmmfetch	HMMDatabase	HMM
ehmmindex	HMMDatabase	HMMDatabase
ehmmpfam	HMMDatabase, Sequence	EhmmpfamOutput
ehmmsearch	HMM, SequenceDatabase	EhmmsearchOutput
emma	MultipleSequence	Alignment, Tree
makenucseq	-	MultipleNucleotideSequence
makeprotseq	-	MultipleProteinSequence
showalign	Alignment	-
showfeat	Sequence	-
showpep	ProteinSequence	-
showseq	NucleotideSequence	-

This table shows the manually defined input and output descriptions for the services in the HMMER subset of the EMBOSS domain.

**Figure 1:**
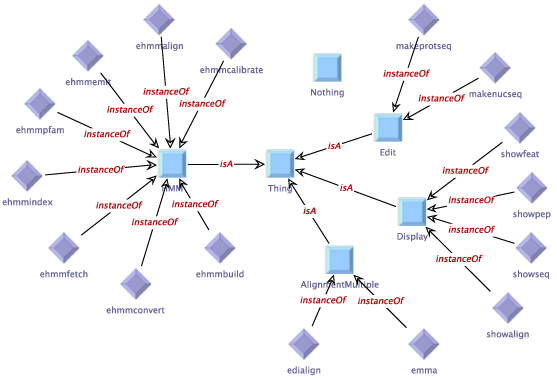
**Manually defined service taxonomy for the HMMER subset of the EMBOSS domain.** This taxonomy contains four abstract groups. Edit has the services makenucseq and makeprotseq as instances, the services showseq, showalign and showtext are classified as Display by the taxonomy. Edialign and emma are abstractly described as AlignmentMultiple, the remaining tools belong to the HMM group.

**Figure 2:**
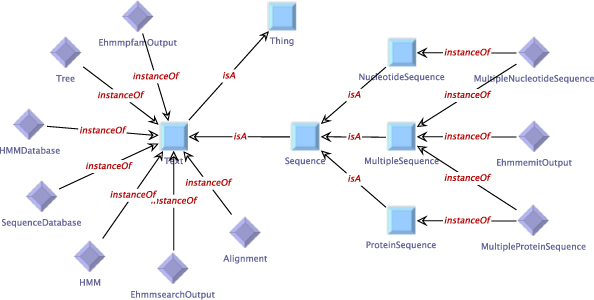
**Manually defined type taxonomy for the HMMER subset of the EMBOSS domain.** As all services in the HMMER subset work on text-based data, all available types in this taxonomy belong to the Text group. The different Sequence types are distinguished further into the groups ProteinSequence, NucleotideSequence, and MultipleSequence. Note that some types are instances of multiple groups: MultipleNucleotideSequence, for instance, is both a MultipleSequence and NucleotideSequence.

Figure 1 shows the manually defined service taxonomy for the HMMER subset, the type taxonomy is given in Figure 2. The (blue) squares in the figure represent the abstract services or types (OWL classes), and the (purple) rhombs are used for concrete instances (OWL individuals). The generic type Thing (center) represents the root of the taxonomy, underneath which abstract groups are defined. The service taxonomy (Figure 1) contains four abstract groups. Edit has the services makenucseq and makeprotseq as instances, and the services showseq, showalign and showtext are classified as Display by the taxonomy. Edialign and emma are abstractly described as AlignmentMultiple, the remaining tools belong to the HMM group. Although it would be natural to classify the HMM tools further (e.g., ehmmalign is also an Alignment service), we leave it this simple for presentation in this paper, as a further classification is not relevant for the given examples. As all services in the HMMER subset work on text-based data, all available types in the type taxonomy (Figure 2) belong to the Text group. The different Sequence types are distinguished further into the groups ProteinSequence, NucleotideSequence, and MultipleSequence. Note that some types are instances of more than one group: MultipleNucleotideSequence, for instance, is both a MultipleSequence and NucleotideSequence.

### Automatic domain setup

In this section we describe how we use different kinds of available meta-information about the tools for the automatic setup of the domain. In short, this setup was created by the followings steps:

1. Generating a skeletal structure for the taxonomies based on the EDAM ontology.

2. Extracting the definition of the input/output behavior from the tools’ ACD files.

3. Linking the services and the determined input/output types to the respective EDAM terms in the taxonomies.

The EMBRACE Ontology for Data and Methods (EDAM, [7]) is an ontology for bioinformatics tools and data, which aims at providing a controlled vocabulary for the diverse services and resources in the life science Semantic Web. The ontology is provided in OBO (Open Biomedical Ontologies) [27] format. Among others, EDAM contains hierarchical term definitions for tool functions and data types, which we use as basis for our service and type taxonomies. The results presented in this paper are based on the EDAM version beta03 (March 2010).

**Figure 3:**
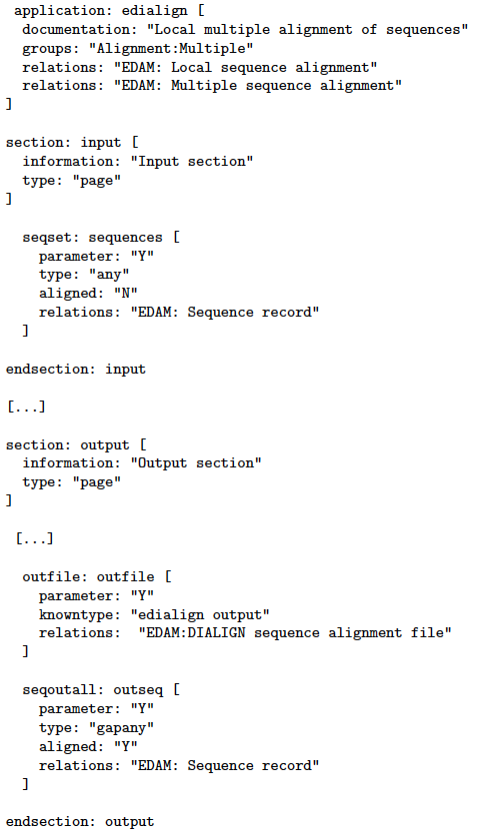
**Example of an ACD file.** ACD file for edialign (slightly shortened).

**Figure 4:**
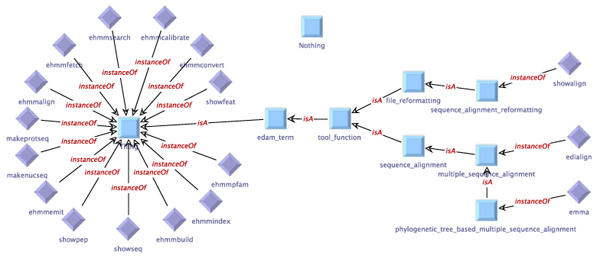
**Automatically created service taxonomy for the HMMER subset of the EMBOSS domain.** In the service taxonomy for the generated domain setup, only three services are classified further in terms of the EDAM Ontology: showalign, edialign, and emma have further classified tool functions. The majority of the services are direct instances of the generic OWL type Thing.

**Figure 5:**
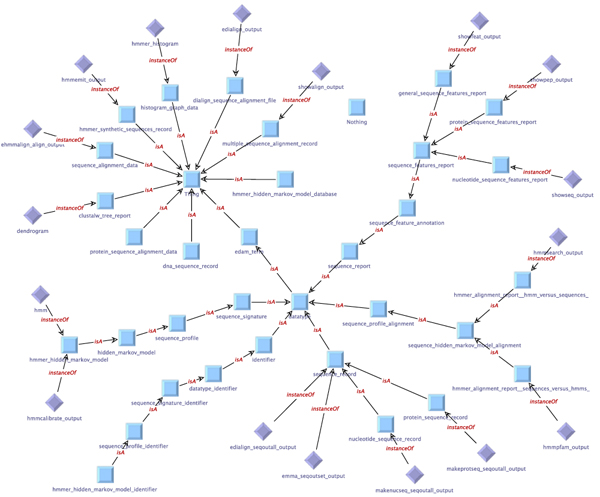
**Automatically created type taxonomy for the HMMER subset of the EMBOSS domain.** The type taxonomy for the generated domain setup contains several EDAM terms for the classification of the various data types. The EDAM terms distinguish, for example , identifiers, sequence_signatures, sequence_records, sequence_reports , and sequence_profile_alignments .

Each EMBOSS tool is accompanied by an ACD (Ajax Command Definition [28]) file that defines its parameters in a special-purpose language. Among plenty other information (the file specifies everything that could be part of the command line invoking the tool or that can be used by another client application), it contains detailed information about the tool’s input and output behavior, including input and output data types, possible other parameters, and indications whether parameters are mandatory or optional. Figure 3 shows a (slightly shortened) ACD file as an example. The first section defines the application’s name (edialign) and the application’s attributes, such as its documentation text and the functional groups that it belongs to. The subsequent sections are used for describing inputs and outputs, where each section can comprise several parameters. In the present example, the input section defines one input parameter (seqset), whereas the output section defines two (outfile and seqoutall). As is also visible from this example, ACD files can contain definitions of relations to EDAM terms. At the time of this writing, around 96% of the available ACD files have already been annotated using EDAM terms, whereby 56% have annotations regarding the application itself, and 95% have parameter annotations. The number of application annotations per file ranges from 1 to 3, with the majority of files providing only one single application relation. The number of parameter annotations varies widely, corresponding to the number of parameters that are defined for the tool (ranging from 1 to 126, at an average of 10 annotations per file). In total, 97% of all parameters are equipped with EDAM relation annotations. We use these annotations to link the individual services and data types to the EDAM terms in our service and type taxonomies. Table 3 lists the services in the HMMER subset of our example domain along with the input/output behavior as derived from the information in the ACD files. Figure 4 shows the service taxonomy for the generated domain setup. In contrast to the manual setup, only three services are classified further in terms of the EDAM Ontology (showalign, edialign, and emma), while the majority of the services remain direct instances of the generic OWL type Thing. The type taxonomy for the generated domain setup (Figure 5) is more comprehensive, containing several EDAM terms for the classification of the various data types. The EDAM terms distinguish, for example, identifiers, sequence_signature s, sequence_records, sequencejreports, and sequence_profile_alignment s , while other types (such as, e.g., sequence_alignment_data and dendrograms are not (yet) consequently covered by the EDAM ontology). These taxonomies reveal that the EDAM Ontology already contains many, but not yet all of the relevant terms that are in frequent use.

**Table 3:** Automatically generated domain: services in the HMMER subset.

Service	Input Types	Output Types
edialign	sequence_record	edialign_seqoutall_output, edialign_output
ehmmalign	protein _sequence_record, hmmer_hidden_markov_model	ehmmalign_align_output
ehmmbuild	protein_sequence_alignment_data	hmm
ehmmcalibrate	hmmer_hidden_markov_model	hmmer.histogram, hmmcalibrate_output
ehmmconvert	hmmer_hidden_markov_model	hmm
ehmmemit	hmmer_hidden_markov_model	hmmemit_output
ehmmfetch	hmmer_hidden_markov_model_identifier, hmmer_hidden_markov_model_database	hmm
ehmmindex	hmmer_hidden_markov_model_database	
ehmmpfam	protein jsequence_record, hmmer_hidden_markov_model	hmmpfam_output
ehmmsearch	protein _sequence_record, hmmer_hidden_markov_model	hmmsearch_output
emma	sequence_record	emma_seqoutset_output, dendrogram
makenucseq	-	makenucseq_seqoutall_output
makeprotseq	-	makeprotseq_seqoutall_output
showalign	sequence _alignment_data	showalign_output
showfeat	sequence_record	showfeat_output
showpep	proteinjsequence_record	showpep_output
showseq	dna_sequence_record	showseq_output

This table shows the automatically derived input and output descriptions for the services in the HMMER subset of the EMBOSS domain.

### Working with the domains

In the previous sections we described the setup of the EMBOSS domain, which is the task of the domain modeler, either by directly defining the domain model (i.e. service descriptions and appropriate type and service taxonomies), or by equipping the services themselves with appropriate meta-information and maintaining ontologies to relate and classify the used terms further, which can be automatically translated into a domain model.

In this section, we illustrate the work of the workflow designer, who develops the actual analysis processes dealing with particular biological questions. Based on the three increasingly complex examples which have been introduced in [19] we show how synthesis problems are specified and what the synthesis methodology derives from these specifications based on the domains described above.

#### Example 1

**Figure 6:**
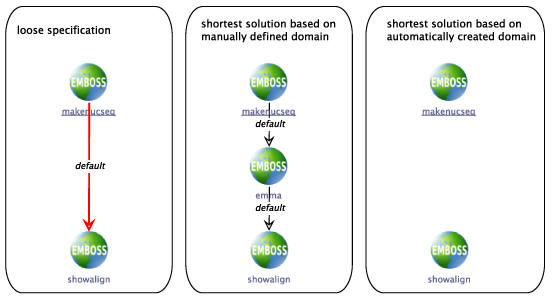
**Synthesis example 1** Loosely specified workflow starting with makenucseq and ending with showalign (left). The synthesis problem is given by the output type of makenucseq, and the input type of showalign. For the manually defined domain, a shortest solution is to insert the multiple alignment algorithm emma (center). For the automatically created domain, no solution can be found (right).

As a first example we consider the small workflow on the left of Figure 6: it consists of the services makenucseq and showalign, which are connected by a loosely specified branch. (For simplicity, we let our example processes begin with services that randomly generate sequences that can be processed further. Note that they can be easily exchanged by the retrieval of sequences from a public database, or by loading a sequence file.) The synthesis problem that is defined by the loose branch is simply given by the output type of makenucseq, providing the input type for the synthesized sequence, and the input type of showalign, which is the type that the synthesized sequence must finally produce.

In case of the manually defined domain, this means that the synthesis algorithm has to find a way from MultipleNucleotideSequence to Alignment. This request can be met by inserting a single multiple sequence alignment service, for example emma: MultipleNucleotideSequence is defined as an instance of MultipleSequence by the type taxonomy (cf. Figure 2), which is emma’s input type (cf. Table 2), while its output type Alignment is directly suitable as input for showalign (cf. Table 2). Figure 6 (center) shows the resulting process.

In case of the automatically created domain, which uses the terminology from the EDAM ontology and the ACD files, the synthesis problem is to find a sequence of services beginning with makenucseq_seqoutall_output and ending with sequence_alignment_data. As Figure 6 (right side) shows, the synthesis does not find a solution for this problem. The reason for this disconnect is that no service in the domain, especially no sequence alignment service, is annotated to produce the type sequence_alignment_data, which is required as input for the showalign services (cf. the service characterizations in Table 3). Rather, the alignment services edialign and emma have output types that are classified as sequence_record (cf. the type taxonomy in Figure 5), so that the synthesis algorithm has no chance to find a possibility to connect them.

#### Example 2

**Figure 7:**
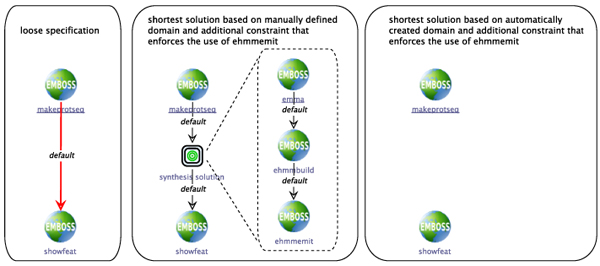
**Synthesis example 2** Loosely specified workflow starting with makeprotseq and ending with showfeat (left). The synthesis problem is given by the output type of makenucseq, and the input type of showfeat. Obviously, the shortest solution is the empty service sequence. Using conditional constraints, it is possible to, e.g., enforce the use of particular services or types. Enforcing the use of ehmmemit leads to inserting a three-step service sequence in case of the manually created domain (center). For the automatically created domain, no solution can be found (right).

A similar synthesis problem is defined by the process shown in Figure 7 (left), where the loosely specified workflow begins with makeprotseq and ends with showfeat, a service that displays features of a sequence. As the output of the first service is a MultipleProteinSequence (manually defined domain) / a makeprotseq_seqoutall_output (automatically created domain), classified by the respective type taxonomies as Sequence / sequence_record, which is the input data type for showfeat, the shortest solution is obviously an empty service sequence (not shown in the figure).

We might, however, have a process in mind that does some analysis on the initially generated sequences and produces another set of sequences, for instance via a Profile HMM. As will be detailed in the Methods section, additional constraints can be used in the workflow specification that is given to the synthesis algorithm. For expressing the sketched case, we can give an additional constraint to the synthesis algorithm that enforces the use of the service ehmmemit.

One of the shortest thus possible processes is shown in Figure 7 (center), obtained by providing the synthesis algorithm with the manually defined domain and an additional constraint that enforced the use of ehmmemit: the initial input sequences are converted into an Alignment by emma, which is then used by ehmmbuild to create a Profile HMM. Ehmmemit emits a set of sequences based on this HMM that are finally displayed by showfeat. The right side of the figure shows the result of a corresponding synthesis run on the automatically created domain, where again no solution can be found. The reason is basically the same as in the previous example: as the alignment services’ outputs are defined as sequence_record rather than as suitable alignment types, the synthesis is not able to recognize them as valid inputs for, e.g., ehmmbuild.

#### Example 3

**Figure 8:**
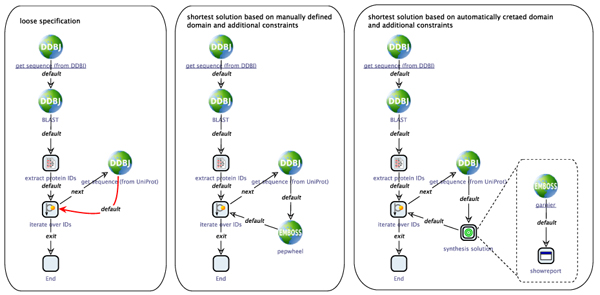
**Synthesis example 3** In this workflow, which does not (yet) contain any EMBOSS services (left), a part of the loop body is a loosely specified and has to be concretized by an appropriate sequence of services. The workflow in the center contains one of the service sequences that were proposed by the synthesis algorithm for the manually created domain and constraints expressing that we want to ”Enforce the use of module Protein2dStructure” and ”Use Display as last service in solution”. The constraints ”Use showreport as last service in solution” and ”Enforce the use of module protein secondary structure prediction ” used together with the automatically created domain leads to the results on the right side of the figure.

As a third and final example in this paper, we discuss the process shown in Figure 8 (left), which does not (yet) contain any EMBOSS services. A (nucleotide) sequence is fetched from the DNA Data Bank of Japan (DDBJ), and used for a BLAST search against a protein database. The Uniprot IDs are extracted from the BLAST result and then processed in a loop that fetches the Uniprot entry for this ID. The remainder of the loop body is a loosely specified branch, to be concretized by an appropriate sequence of services. The synthesis plugin has access to both the EMBOSS and the DDBJ domain model and can transparently combine services from both sources. For this example, we did not only use the HMMER subset but the complete EMBOSS domains to find an appropriate sequence of services that does something with the protein sequence that is retrieved within the loop.

If we start the synthesis with no further constraints, thousands of possible solutions are found, even if the length of the solution is limited. The reason lies in the nature of the EMBOSS domain: many tools work on very similar input types (sequence), some again producing sequences, so that if the synthesis is only based on the type information, unfathomable many variations of solutions are possible.

Thus we refine our specification and formulate additional constraints for the synthesis in order to get less, but more reasonable results. For instance, we might want the inserted service sequence to end with a service that visualizes a result in some fashion, possibly after having applied some analysis to the sequence. The center of Figure 8 shows the workflow with one of the service sequences that were proposed by the synthesis algorithm for the manually created domain and constraints expressing that we want to “Enforce the use of module Protein2dStructure” and “Use Display as last service in solution”, where Protein2dStructure and Display are abstract service groups. This request is met, for instance, by pepwheel, a service that draws a helical wheel diagram for a protein sequence.

For the automatically created domain, where the EDAM terminology is used for the constraint formulation, we use the constraints “Use showreport as last service in solution” (showreport being a concrete service) and “Enforce the use of module protein secondary structure prediction” (abstract service), as there are no EDAM terms that directly correspond to the abstract service groups that we defined in the manual domain setup. The right side of Figure 8 shows one of the possible results of this synthesis run, where the services garnier (a service predicting protein secondary structures) and showreport (simply displaying the textual content of, e.g., the EMBOSS report that is produced by garnier) have been inserted. The different constraints and the different corresponding results that we encounter in this example show that not only the process specification and the resulting service sequences, but also the constraint formulation itself (as part of the specification) depend on the concrete structure of the domain model.

## Conclusions

Our experiments demonstrate that comprehensive domain models in combination with adequate synthesis methodology greatly simplify working with the large, heterogeneous, and hence manually intractable EMBOSS collection. However, they also show that with the information that can be derived from the (current) ACD files and EDAM ontology alone, some essential connections between services cannot be recognized. A striking example is the disconnect between the alignment services (e.g., edialign, emma) and alignment visualizers such as showalign. Due to the reason that the alignment services’ outputs are simply described as sequence records whereas some kind of sequence alignment data would make a suitable input for showalign, an artificial separation of actually compatible types has been introduced. This reveals that although the descriptions of the individual components are technically sound and several ontological terms are well defined, they are not (yet) sufficiently synchronized with respect to the automatic construction of executable workflows. Thus, automatically created domain models should be manually revised in order to detect and bridge essential gaps.

Clearly, adequate domain modeling requires to incorporate as much domain knowledge as possible, far beyond the mere technical aspects of the different types and services. Finding or defining semantically appropriate service and type descriptions is a difficult task [29], which is common among all approaches to (semi-) automatically dealing with the large number of distributed, heterogeneous services that are available in the bioinformatics application domain. Projects like BioMoby [3], SADI [4], BioCatalogue [5], the (my)Grid Ontology [6], and the EDAM Ontology address this issue by providing knowledge bases that particularly capture bioinformatics data types and services. We plan to integrate (more of) their services and domain knowledge in the scope of future case studies with Bio-jETI and PROPHETS. The resulting domains will contain far more heterogeneous services than the comparatively ’closed’ EMBOSS domain that we used for the current study, creating new challenges for the client-side software, challenges that our methods are designed for.

## Methods

Bio-jETI [17,30] is a framework for model-based, graphical design, execution and management of bioinformatics analysis processes. It has been used in a number of different bioinformatics projects [31-34] and is continuously evolving as new service libraries and service and software technologies become established.

Technically, Bio-jETI is based on the jABC modeling framework [35] as an intuitive, graphical user interface and the jETI electronic tool integration platform [36] for dealing with remote services. Using the jABC technology, process models are constructed graphically by placing services on a canvas and connecting them according to the flow of control. jABC process models are directly executable by an interpreter component, and they can be compiled into a variety of target languages via the Genesys code generation framework [37].

In [18], we presented our approach to semantics-based service composition in the Bio-jETI platform. By integration of automatic service composition functionality into an intuitive, graphical process management framework, we maintained the usability of the latter for semantically aware workflow development. Furthermore, we could integrate services and domain knowledge from any kind of heterogeneous resource at any location, and were not restricted to any semantically annotated services of a particular platform. For the work presented in this paper, we used the PROPHETS (Process Realization and Optimization Platform using a Human-readable Expression of Temporal-logic Synthesis) extension of the Bio-jETI platform that simplifies workflow development in order to even reach biologists without programming background. PROPHETS seamlessly integrates automatic service composition into the jABC. It enhances the previous approaches by including more formal methodology, but with less of it being required for the user to know, thus enabling the system to be used by a wider range of users. These enhancement are in particular:

● visualized/graphical semantic domain modeling.

● loose specification within the process model.

● non-formal specification of constraints using natural language templates, and

● automatic generation of model checking formulas (to check global properties processes).

Two roles are designed for using this extension. The* domain modeler* provides information on available services and a semantic classification of these services and their input and output types. The* workflow designer* is the one who uses the available services to model the processes. The following two subsections deal with one of those roles, respectively, while the subsequent ones give more detail on the synthesis method and verification concerns.

### Domain modeling

The basis of the domain model is built by meta-information on services, which enhances the definition of jABC services regarding their abstract input/output behavior. Throughout our framework types are represented by symbolic names, thus abstracting from concrete implementations. Each service is characterized by two subsets of the set of all symbolic type names, namely* input types* and* output types.* The meta-information is stored as a separate file within the current project’s directory, which allows for the usage of a specialized nomenclature for different jABC projects, even though the included services might be the same.

Furthermore, the services and types can be classified using taxonomies. These taxonomies are expressed as ontologies in OWL format, where the concept* Thing* denotes the most general type or service, respectively. Using the* is-a* relation, additional semantic classifications can be added into the domain. The actual types and services are then represented as individuals that are related to one or more of those classifications by the* instance-of* relation. Although we also provide a seamlessly integrated graphical editor for these OWL files (Figures 1,2, 4 and 5), the domain modeler may use any OWL tool of his preference.

Finally, there might be domain specific knowledge like ordering constraints on services or general compatibility information. This knowledge must be formalized appropriately. Basically, there are two possible options to do so: Either the domain expert expresses model checking formulas that must hold for every process within the project, or he defines global constraints that are used for every synthesis. Furthermore, the system that allows formulae to be expressed with natural language templates, can be extended to the needs of the specific domain.

### Process design

**Figure 9:**
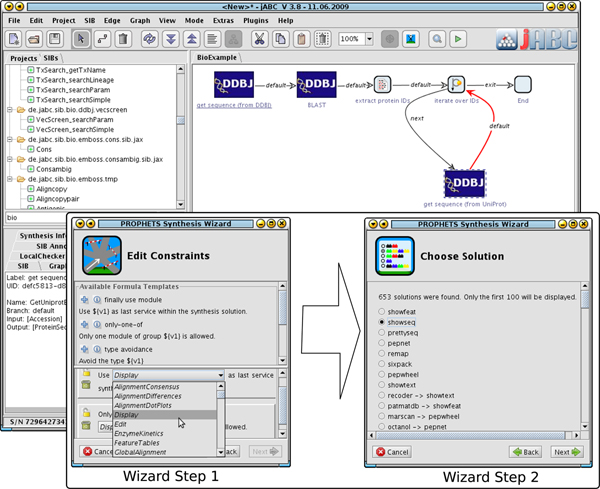
**Synthesis Execution** Shows a loosely specified process (background, cf. Figure 8 - Synthesis example 3) and the wizard windows (foreground) that query the user for additional input. Step 1 shows the constraint editor that is based on natural language templates, and in Step 2, the user can choose one out of all the solutions that the synthesis algorithm found.

After a domain has been set up by the domain expert, it can be used by the workflow designer to model processes. As part of the seamless integration into the jABC, PROPHETS concentrates on the usability for non-technical users. It mainly differs from our previous synthesis approaches [18] in the idea of* loose specification*: branches in the model can be marked as* loosely specified,* which then automatically are replaced by reasonable services by our framework. Therefore, the process designer neither needs to model fully executable processes (the standard Bio-jETI way) nor formally specify a synthesis or planning problem with some first-order or temporal logic. Behind the scenes the algorithm still requires formal specifications of the synthesis problem, but our goal with the here presented approach is to hide this formal complexity from the user and replace it by intuitive (graphical) modeling concepts. Furthermore, the actual execution of the synthesis is presented to the user as a set of wizard windows where he finally can choose the favored solution from the list of all possible solutions (”Wizard Step 2” in Figure 9). 

Each loosely specified branch’s synthesis can be enhanced by additional constraints. As we will not expect common process designers to deal with this formal specification, we provide means to express constraints using a system that is based on templates in natural language. The user chooses a restricting concept and then simply has to fill in a cloze text with prepared values ("Wizard Step 1” in Figure 9). The possible values for the cloze text fields are automatically extracted from the domain (i.e. service definition and taxonomies).

### Synthesis algorithm

The algorithm [38] that we use to complete a loosely specified process to be fully executable takes two aspects into account: On the one hand, the process must be a valid execution regarding type consistency, on the other hand, the constraints specified by the process designer must be met.

**Figure 10:**
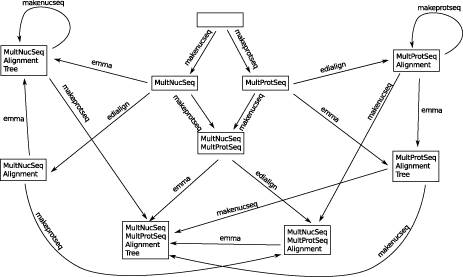
**Synthesis Algorithm’s Configuration Universe** Small excerpt of the search space that is used by the synthesis algorithm to find possible solutions. The nodes represent available (i.e. already generated) types, while the edges are the services that create new ones.

The* configuration universe* constitutes the algorithm’s basic search space. It contains all valid execution sequences and is implicitly defined by the domain model as follows: Each subset of the overall type set denotes a state. The edges represent state transformations caused by the execution of services. An edge is inserted for a service, if the input types of the service are a subset of the types at the edge’s originating state and the target state is the union of the service’s output types and the original types. As this configuration universe usually is very large, it is not explicitly generated from the domain definition, but on the fly within the synthesis process. Figure 10 shows a small excerpt of the configuration universe for the example domain that we use in this paper. To maintain the readability, only 4 of the full example domain’s (which was already simplified by only containing 17 out of over 350 services) services are included in the figure.

The* specification formula* is the second aspect. It describes all sequences of services that meet the individual workflow specification, but without taking care of actual executability concerns. As the explicit representation of all those possible sequences might be extremely large, it also is not explicitly built, but given declarative as a formula in SLTL (Semantic Linear Time Logic) [38], an extension of the commonly known propositional linear-time logic (PLTL). This formula is created by conjunction of all constraints, i.e. the constraints that are specified by the process designer for the current loosely specified branch and the ones that were globally specified by the domain modeler.

To start the search for solutions, the synthesis algorithm requires an initial state (i.e. a set of start types). In contrast to our previous approach [18], where these start types had to be specified manually, they are now determined automatically according to preceding services using data-flow analysis methods. The types that are created on the execution path from the workflow’s initial node to the currently synthesized loosely specified branch are taken as start types. If due to branching in the model multiple paths are possible, the largest set of types that is consistent with each of those paths is taken.

Given these specifications, the synthesis algorithm performs a parallel evaluation of the configuration universe and the specification formula to search for paths that are consistent with the configuration universe and fulfill the SLTL formula. Each of those paths is a valid solution that may replace the loosely specified branch. The framework currently supports two possibilities to choose one of the solutions: Either the shortest solution is chosen automatically or the user is queried to select one. However, the general architecture of the framework allows for the easy integration of other solution choosing mechanisms, for instance based on some cost function in order to obtain the* cheapest* solution.

### Verification

**Figure 11:**
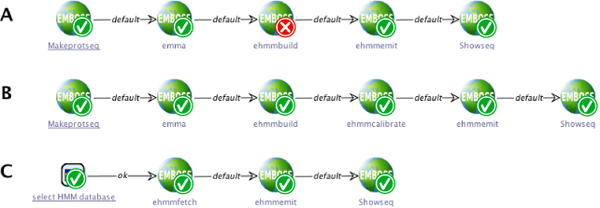
**Model checking examples.** Model checking of a formula expressing that each built HMM must be calibrated before it actually emits sequences.

As already mentioned, the domain modeler can define high-level constraints using model checking [39] formulas to express properties that must hold for any model in this domain. For an example, consider the process in Figure 11(A), where a HMM is built from a multiple sequence alignment (obtained via emma) and used to produce sequences that are finally displayed using showseq. The workflow designer now might want to express that each built HMM has to be calibrated before it actually emits sequences. Formally, this can be expressed with the PLTL formula *ehmmbuild * (*ehmmemit*** WU***ehmmcalibrate*) denoting that the use of ehmmbuild implies that ehmmemit is not used before ehmmcalibrate has been executed. As Figure 11(A) shows, this requirement is not met by the previously created process, because at the ehmmbuild service’s node, the property is not fulfilled (indicated by the red ”x” overlay icon in the lower right corner). Inserting the ehmmcalibrate service into the workflow fixes this issue, as Figure 11(B) shows: all services are marked by a green icon. Naturally, and as 11(C) shows, this constraint is also fulfilled if the HMM is not built by the process, but fetched from an HMM database.

## List of abbreviations used

**ACD:** Ajax Command Definition;** API:** Application Programming Interface;** BLAST:** Basic Local Alignment Search Tool;** DDBJ:** DNA Data Bank of Japan;** EDAM:** EMBRACE Data And Methods ontology;** EMBOSS:** European Molecular Biology Open Software Suite;** GUI:** Graphical User Interface; **HMM:** Hidden Markov Model;** HTN:** Hierarchical Task Network;** ID:** Identifier;** jABC:** Java Application Building Center;** jETI:** Java Electronic Tool Integration;** OBO:** Open Biomedical Ontologies;** OWL:** Web Ontology Language;** PLTL:** Propositional Linear Time Logic;** PROPHETS:** Process Realization and Optimization Platform using a Human-readable Expression of Temporal-logic Synthesis;** SADI:** Semantic Automated Discovery and Integration;** SLTL:** Semantic Linear Time Logic

## Competing interests

The authors declare that they have no competing interests.

## Authors' contributions

AL developed the presented examples and drafted the manuscript. SN designed and implemented the PROPHETS plugin and contributed essential parts to the manuscript, especially to the Methods section. TM and BS have been developing the concept of the jABC and jETI platforms since 1993, first in the area of formal verification tools, then in the area of Semantic Web services. They have revised and edited the manuscript. All authors read and approved the final manuscript.
